# Transformation from Electromagnetic Inflection to Absorption of Silicone Rubber and Accordion-Shaped Ti_3_C_2_MXene Composites by Highly Electric Conductive Multi-Walled Carbon Nanotubes

**DOI:** 10.3390/polym15102332

**Published:** 2023-05-17

**Authors:** Xin Guo, Li Liu, Naixiu Ding, Guangye Liu

**Affiliations:** Engineering Research Center of High-Performance Polymer and Molding Technology, Ministry of Education, Qingdao University of Science and Technology, Qingdao 266042, China; 0018020008@mails.qust.edu.cn (X.G.); liuli@qust.edu.cn (L.L.)

**Keywords:** electromagnetic inflection, electromagnetic wave absorption, Ti_3_C_2_MXenes, highly electric conductive multi-walled carbon nanotubes

## Abstract

Electromagnetic (EM) pollution becomes more penetrating in daily life and work due to more convenience provided by multi-electrical devices, as does secondary pollution caused by electromagnetic reflection. EM wave absorption material with less reflection is a good solution to absorb unavoidable EM radiation or reduce it from the source. Filled with two-dimensional Ti_3_SiC_2_MXenes, silicone rubber (SR)composite demonstrated a good electromagnetic shielding effectiveness of 20 dB in the X band by melt-mixing processes for good conductivity of more than 10^−3^ S/cm and displayed dielectric properties and a low magnetic permeability; however, the reflection loss was only −4 dB. By the combination of one-dimensional highly electric conductive multi-walled carbon nanotubes (HEMWCNTs) and MXenes, the composites achieved the transformation from electromagnetic inflection to an excellent absorbing performance to reach a minimum reflection loss of −30.19 dB due to electric conductivity of above 10^−4^ S/cm, a higher dielectric constant, and more loss in both dielectric and magnetic properties. Ni-added multi-walled carbon nanotubes were not able to achieve the transformation. The as-prepared SR/HEMWCNT/MXene composites have potential application prospects in protective layers, which can be used for electromagnetic wave absorption, electromagnetic interference suppression of devices, and stealth of the equipment.

## 1. Introduction

Silicone rubber-based composites have been studied in recent decades to develop lightweight electromagnetic (EM) shielding materials with enhanced electrical and thermal properties to provide solutions for electromagnetic interference (EMI) pollution. The development of flexible EMI shielding sheets are of great importance for more and more electromagnetic radiation occurring following the fast-growing miniaturized and complex electronics with integrated circuits and electronic devices, such as micro-robots and wearable devices. The traditional EM shielding materials filled with metal are too heavy to wear and are not flexible to suit a variety of equipment and product shapes, although they are used to attenuate electromagnetic radiation due to their high electrical conductivity. Silicone rubber composites containing electrical fillers are considered to be an excellent alternative to traditional materials in view of their flexibility and process efficiency.

The EMI functionalization of silicone rubber composites has been realized by adding a large number of conductive fillers or magnetic fillers for its low processing viscosity and high filling ability. Carbon materials are widely used in the EMI materials, such as carbon black, graphene [1], carbon fibers [2], and carbon nanotubes, etc. Multi-walled carbon nanotubes (MWCNTs) are widely used in the field of electromagnetic shielding and wave absorbing composite materials because of their considerable ability to induce important changes in various properties. In the application of EM shielding rubber composites, carbon nanotubes can not only be used as reinforcing fillers but can also form electron flow paths and EM shielding networks in the matrix. MWCNTs and Ti_3_C_2_MXenes are attarcting fillers for excellent EMI and wave absorbing composites. Under the action of alternating electromagnetic fields, carbon nanotubes can be equivalent to dipoles and generate dissipative current, which makes electromagnetic wave energy dissipate by heat energy and exert wave absorbing performance, and therefore, they are applied in the application fields of dielectric material [3], and electric conductive material [4] (EMI) shielding materials [5,6,7,8,9,10,11,12]. With unique performances, such as a high conductivity (up to 24,000 S/cm), a high Young’s modulus (up to 380 GPa), flexibility, and tunable and hydrophilic surfaces [13], MXenes have been studied and applied in various fields, including energy storage and conversion [14], electrocatalytic, sensing [15,16], electromagnetic interference shielding [17,18], microwave absorption [19], wireless communication [20], structural materials, friction material, repair, and biomedical fields [21], as well as Ni-contented fillers [22]. It has been demonstrated that MXene (6 wt%)/EPDM with 0.3-mm thick exhibits EMI shielding performances up to 48 dB in the X-band (8.2 to 12.4 GHz) and 52 dB in the Ku-band (12.4 to18 GHz) when the MXene content is 6 wt%, respectively [23].An efficient vacuum-assisted filtration approach was studied for the fabrication of flexible and highly conductive Ti_3_C_2_TxMXene/natural rubber (NR) nanocomposites by constructing interconnected MXene networks in the NR matrix. The as-prepared NR/MXene nanocomposite with 6.71 vol % of MXenes showed a superb electrical conductivity of 1400 S/m and an excellent EMI shielding performance of 53.6 dB [24]. 

The EMI shielding effectiveness of these composites is much better than that of other rubber blends; however, the EM wave absorbing performance is insufficient. Electromagnetic wave absorbing material is a specific type of EMI material that can be used to protect human beings and precise devices from hazardous interference by electromagnetic waves, as well as reduce second radiation pollution by reflection spontaneously. However, it has not been widely concerned to be designed with a dominant contribution from absorption rather than EM wave reflection. In this work, one-dimensional highly electric conductive multi-walled carbon nanotubes were used to realize transformation from the electromagnetic reflection of silicone rubber composites with Ti_3_C_2_MXenes to absorption. An approach was proposed to achieve this transformation, and a possible construction design might be the solution to maintain good EMI shielding effectiveness and an excellent wave absorbing performance. The as-prepared silicone rubber/ HEMWCNT/MXene composites show potential in the application field of EM wave absorption in the X-band. 

## 2. Materials and Methods

### 2.1. Materials

Mixed silicone rubber of model 6140 (shore A hardness of 40) was purchased from Shandong Dongyue Silicone Materials Co., Ltd. (Zibo, China). The 2, 5-dimethyl-2, 5-bis (tert-butyl peroxide) hexane with an effective content of 50% was purchased from Shanghai Bojing Chemistry Co., Ltd. (Shanghai, China). Highly electric conductive MWCNTs of the Model TNEM3 was 10 nm to 20 nm in diameter and less than 30 μm in length with the purity of more than 90%. Nickel-added multi-walled carbon nanotubes (NiMWCNTs) TNNiM8 were 30 nm to 80 nm in diameter and less than 10 μm in length, with a nickel content of more than 60 wt% and CNTs content of more than 38 wt%, respectively. Both TNEM3 and TNNiM8 were purchased from Chengdu Zhongke Times Nanoenergy Materials Ltd. (Chengdu, China). The two-dimensional transition metal oxide Ti_3_C_2_MXenes were purchased from Suzhou Beike Nano Technology Co., Ltd. (Suzhou, China) with the multi-layered accordion shape prepared by the organic-basic intercalation agent (dimethyl sulfoxide, etc.)

### 2.2. Preparation of Mixed Silicone Rubber/Multi-Walled Carbon Nanotube/MXene Composites

The sample SR/10MX was mixed by melt mixing in an open two-roll mill according to 100 phr (per hundred rubbers in weight portions) silicone rubber, 2 phr 2, 5-dimethyl-2, 5-bis (tert-butyl peroxide) hexane, and 10 phr Ti_3_C_2_MXenes, and the mixed compound was vulcanized in a vulcanizing press at 170 °C for 6 min, and then underwent a second vulcanization in an oven for further cross-linking at 200 °C for 8 h. The sample SR/10NiMWCNT used 10 phr NiMWCNTs to replace the MXenes.

The difference between the sample SR/10MX10HEMWCNTs and the SR/MX was one more filler of 10 phr HEMWCNTs loaded into the formula. For SR/10MX10NiMWCNT composites, the HEMWCNTs were replaced by the NiMWCNTs. And the cure time for these compound was 2 min longer than t_90_.

### 2.3. Test Instruments and Methods

Vulcanization parameters of the composites were measured by RPA2000 (Alpha Technology Inc., Bellingham, WA, USA) at 170 °C for 20 min in accordance with ISO 3417:1991 Rubber—Measurement of vulcanization characteristics with the oscillating-disc curemeter. Tensile properties of the samples were tested at a tensile rate of 500 mm/min via the tensile tester (AI-7000S, GOTECH testing machine corporation, Taiwan, China) in accordance with ISO 37:2005 Rubber, vulcanized, or thermoplastic—Determination of tensile stress-strain properties. Dynamic properties of the unvulcanized sheets were conducted on the RPA2000 tester at the temperature of 100 °C with a strain range from 0% to 100% at the frequency of 1 Hz, in accordance with the ASTM D 6204 Test Method for Rubber—Measurement of unvulcanized rheological properties using rotorless shear rheometers. Heat resistance and thermal stability of the composites were tested by a thermal gravimetric analyzer (TG209F1, NETZSCH, Bavaria, Germany) from 30 °C to 800 °C at the heating rate of 20 °C/min under nitrogen (N_2_). The infrared spectrometer (TENSOR 27, BRUKER, Broekselle, Germany) was used to characterize the structure of the composites by scanning 32 times from 500 cm^−1^ to 4000 cm^−1^ in the mode of ATR (attenuated total internal reflection). The electrical properties of the composites were measured by a broadband dielectric impedance spectrometer (NOVOCONTROL, Beijing Huidexin Technology Co., Beijing, China) after the samples were cut into round sheets with a diameter of 25 mm, and both sides were sprayed gold by the SBC-12 small ion sputtering instrument (Beijing Zhongke Keyi Co., Ltd., Beijing, China). The electromagnetic interference shielding effectiveness was measured by the vector network scanner with waveguide clips (R&S ZNB20, SCHWARZ, Baden-Württemberg, Germany) from 8200 MHz to 12,400 MHz in a cuboid specimen of 22.86 mm in length, 10.86 mm in width, and an actual thickness measured by a vernier caliper. The real part ε′ and the imaginary part ε″ of the dielectric constant, and the real part μ′ and the imaginary part μ″ of the magnetic permeability of the composites were all measured by a vector network scanner as mentioned above. As the characterization parameter of microwave absorbing performance, the reflection loss was calculated by the Matrix Lab software (Matlab R2022b, The MathWorks, Inc., Alpharetta, GA, USA) corresponding to the simulated matching thickness and frequency of the incident wave as according to the real part ε′ and the imaginary part ε″ of the dielectric constant, and the real part μ′ and the imaginary part μ″ of the magnetic permeability.

## 3. Results and Discussion

### 3.1. Vulcanization Parameters

The vulcanization parameters of the silicone rubber/MWCNT/MXene composites are shown in Table 1. With 10 phr Ti_3_C_2_MXenes, the highest torque MH of the SR/10MX was 6 dNm lower than that of the SR/10NiMWCNTs with 10 phr nickel-added multi-walled carbon nanotubes. The difference between the MH and the lowest torque ML of SR/10MX was 12 dNm, indicating a lower cross-linking degree formed in the materials when the Ti_3_C_2_MXenes were used compared with the nickel-added multi-walled carbon nanotubes, due to the effect on the vulcanization heat transfer of materials by the layered structure of MXenes. The optimum vulcanization time (t_90_ + 2 in minute) of this sample was 6 min, which was the longest in this group of samples. It fully confirms the correctness of the above phenomena that nickel-added multi-walled carbon nanotubes show good thermal conductivity when the nickel content was over 60%, hence the vulcanization time of the sample SR/10NiMWCNTs and SR/10MX10NiMWCNTs was 4.2 min and 3.58 min, respectively. At the same time, nickel-added multi-walled carbon nanotubes presented a higher density due to their large amount of metal elements, which also improved the resistance to shear deformation and torque to a certain extent. The MH-ML values of the samples reflect the degree of cross-linking which affects the tensile strength of the materials. The higher the MH-ML values of the samples, the higher the crosslinking degree is, resulting in the higher tensile strength.

### 3.2. Dynamic Mechanical Properties

**Figure 1:**
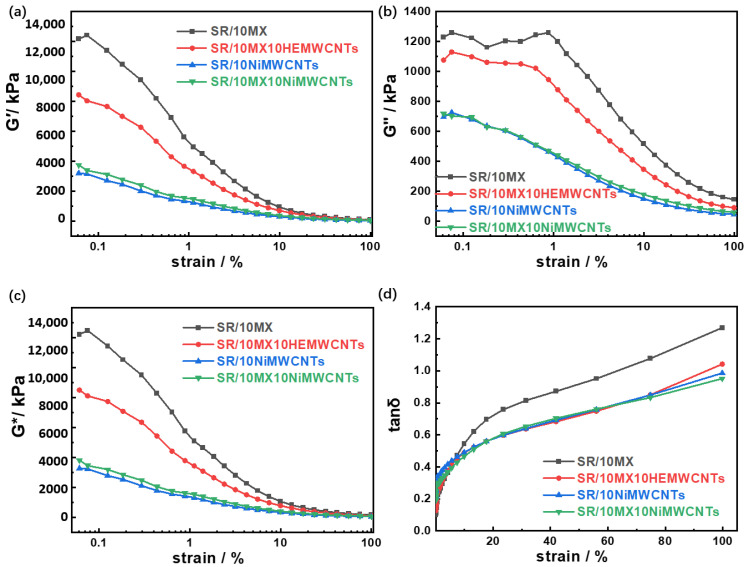
Dynamic mechanical properties of the silicone rubber/multi-walled carbon nanotubes /MXene composites: (**a**) elastic modulus; (**b**) loss modulus; (**c**) composite modulus; and (**d**) tanδ corresponding to the strain change from 0% to 100%.

As shown in Figure 1a, the initial elastic modulus of the SR/10MX composite filled with Ti_3_C_2_MXenes was able to reach 12,174 kPa at a low strain, which was three times that of the SR/10NiMWCNTs and the SR/10MX10NiMWCNTs filled with nickel-added multi-walled carbon nanotubes. This was because Ti_3_C_2_MXenes with the two-dimensional layered structure contributes elastic modulus subjected to small shear strain. With the highly electric conductive carbon nanotubes and MXenes, the elastic modulus of the SR/10MX10HEMWCNT composites were twice that of the SR/10NiMWCNTs and the SR/10MX10NiMWCNTs. The length of the nickel-added multi-walled carbon nanotubes was 10 μm, while the length of highly electric conductive multi-walled carbon nanotubes was about 30 μm. The latter was three times as long as the former in length, and it was easier to form physical entanglements with each other [25], which thereby contributed to the physical cross-linking density, and showed a higher elastic modulus. The physical entanglement ratio of the materials increases with significant Payne effect. The elastic modulus G′ of all the samples decreased nonlinearly with the increase of shear strain. The uniform and continuous distribution of the highly electric conductive multi-walled carbon nanotube network was the main contributor to the elastic modulus of the SR/10MX10HEMWCNT composites under low shear strain. However, the network of carbon nanotubes network was destroyed, and the silicone rubber matrix became the main contributor to the elastic modulus G′ when the samples were subjected to high shear strain. Under high shear strain, the elastic modulus of the four materials was found to be similar as the matrix of the materials was the same silicone rubber.

As shown in Figure 1b, the loss modulus of the SR/10MX decreased greatly with the increaseof shear strain and became lower at high strain than that of the samples with the highly electric conductive multi-walled carbon nanotubes. The difference of the loss modulus of the four samples was mainly caused by vulcanization degree, which was influenced by the heat transfer of the fillers. The higher the degree of cross-linking, the smaller the distance between the cross-linking points, the greater the rigidity of the composites, and the faster the response speed to strain with less loss. With a combination of MXenes and HEMWCNTs, or Mxenes only, the composites show a lower cross-linking degree, leading to more terminal groups of molecular chains with more loss, hysteresis loss, and a higher loss factor, which are consistent with those shown in Figure 1d.

As shown in Figure 1c, the reason of the similar composite modulus and elastic modulus was the same as that of with elastic modulus for four silicone rubber/highly conductive multi-walled carbon nanotube/Mxene composites. 

As shown in Figure 1d, the loss factors of the silicone rubber/HEMWCNT/Mxene composites varied in the strain range from 0 to 100%, which was closely related to the response speed of the network built by cross-linking or entanglements. The damping factor of SR/10MX was higher than 0.3 in a wide strain range. However, the rigidity of the SR/10MX10HEMWCNT composites increased, resulting in a faster response to the strain, and the loss of the composites was decreased due to the cross-linking contribution of physical entanglement after adding the carbon nanotubes.

### 3.3. Thermal Behavior

**Figure 2:**
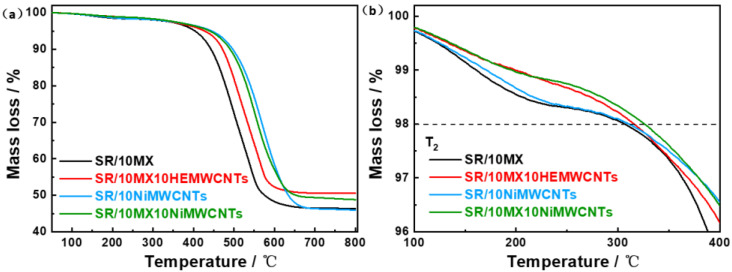
Thermal behavior of the silicone rubber/multi-walled carbon nanotubes/Mxene composites in N_2_ from 50 °C to 800 °C: (**a**) thermal gravimetric analysis curve; and (**b**) temperature corresponding to mass loss of 2%.

The high temperature resistance and ash content of silicone rubber/multi-walled carbon nanotubes/Mxene composites were tested by the thermogravimetric tester, as shown in Figure 2a. To compare heat resistance, temperature corresponding to 2% weight loss is shown in Figure 2b. Filled with Mxenes, the samples SR/10MX10HEMWCNTs and SR/10MX10NiMWCNTs lost 2% weight at 316 °C and 327 °C, respectively, which were 10 °C and 17 °C higher than the other two samples, respectively. These materials contained more electric conductive fillers which degraded at a high temperature, while the ash content was calculated as 50.5% and 48.9% respectively, partly from the residual metal after the Mxenes were degraded. The decomposition residues of the SR/10MX and SR/10NiMWCNTs were 46.4% and 46.2%, respectively. The overall residues were higher due to the residual carbon and metal elements in the samples during thermal weight loss in nitrogen.

**Figure 3:**
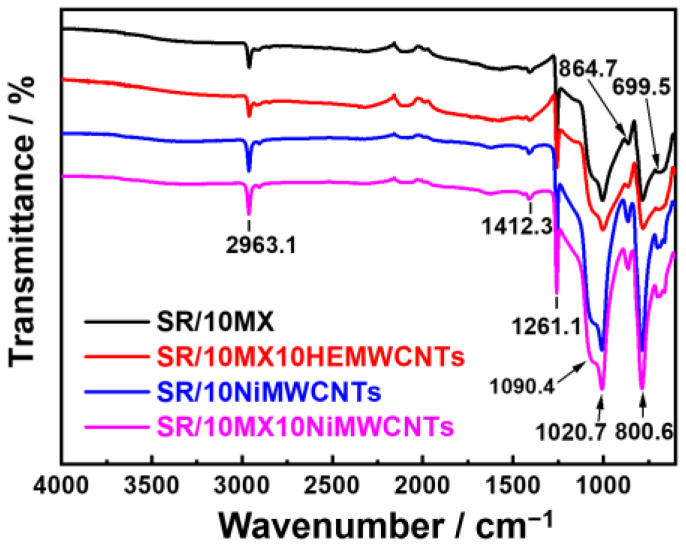
The attenuated total reflection infrared spectra of the silicone rubber/multi-walled carbon nanotube/MXene composites.

To further confirm the reasons for the observed differences in temperature resistance of these composites, the samples were tested by attenuation total reflection infrared (see Figure 3) The peak of 699.5 cm^−1^ was attributed to the bending vibration of Si-O bond; the peak of 800.6 cm^−1^ was ascribed to the in-plane bending vibration of the CH_3_ groups and the Si-C bond stretching vibration, where the Si atom connects with one-to-two methyl groups; the peak of 864.7 cm^−1^ was attributed to the in-plane bending of the CH_3_ group and the out-of-plane bending of C-H bond on the olefins; the peak of 1020.7 cm^−1^ was attributed to the stretching vibration of the Si-C bond; the peak of 1090.4 cm^−1^ was attributed to the stretching vibration of the Si-O bond and the strong absorption band of Si-O-Si; the peak of 1261.1 cm^−1^ was attributed to the symmetric deformation vibration of the CH_3_ groups directly connected with Si; the peak of 1412.3 cm^−1^ was attributed to the anti-symmetric deformation vibration of the CH_3_ group directly connected to Si; and the peak of 2963.2 cm^−1^ was attributed to the stretching vibration of the C-H bond.

The infrared spectra of these composites presented the same position and similar intensity among the samples. MXenes, nickel-added multi-walled carbon nanotubes, and highly electric conductive multi-walled carbon nanotubes did not change the chemical structure of the composites. The thermal behavior mainly reflects the influence of the carbon nanotubes and the MXenes.

### 3.4. Morphological Structure

**Figure 4:**
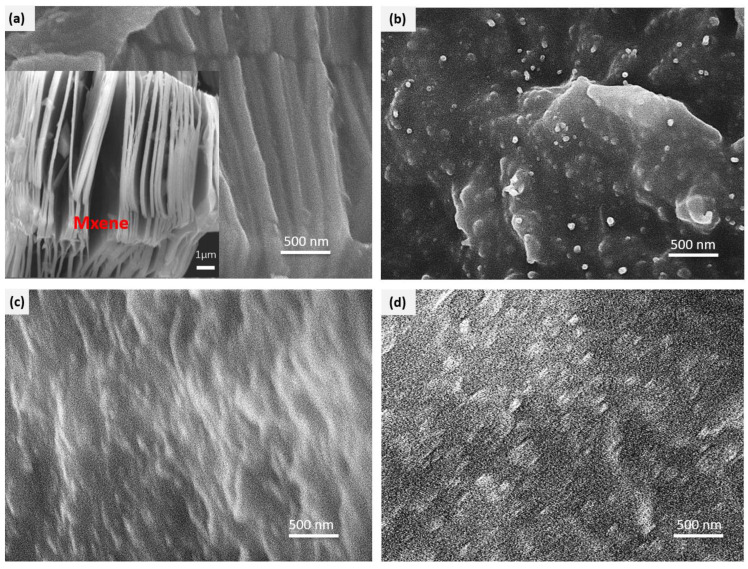
SEM images of the silicone rubber/multi-walled carbon nanotube/MXene composites at the scale of 500 nm and the magnification of 50,000×:(**a**) SR/10MX with a subfigure of accordion-shaped morphological SEM of Ti_3_C_2_MXenes; (**b**) SR/10MX10HEMW CNTs; (**c**) SR/10NiMWCNTs; and (**d**) SR/10MX10NiMWCNTs.

The accordion-like structure of the Ti_3_C_2_MXenes was maintained as the same which can be observed in the cross-section of the silicone rubber/MXene composites from the SEM image of SR/10MX, as shown in Figure 4a.

Combined with the accordion-like morphology and scale of Ti_3_C_2_MXenes material in the subfigure of Figure 4a, it was determined that the accordion-like cross-section came from the MXene fillers when the MXenes were added to the silicone rubber. However, the cross-section of the material with MXenes and HEMWCNTs was dominated by uniformly distributed multi-walled carbon nanotubes, while some MXenes sheets were seen, as shown in Figure 4b. The uniform distribution of nickel-added multi-walled carbon nanotubes was observed in the cross-sections of the silicone rubber composites with NiMWCNTs in Figure 4c, and with both MXenes and NiMWCNTs shown in Figure 4d.

With good compatibility between the MWCNTs and the silicone rubber, a good dispersion state of the composites was achieved by melt mixing. HEMWCNTs benefited the distribution of the MXenes after being exfoliated by the one-dimensional HEMWCNTs, which was different from the NiMWCNTs. High electric conductive multi-walled carbon nanotubes have a significant impact on the morphology of the composites, which is supposed to have a greater impact on the properties.

### 3.5. Dielectric and Magnetic Properties

**Figure 5:**
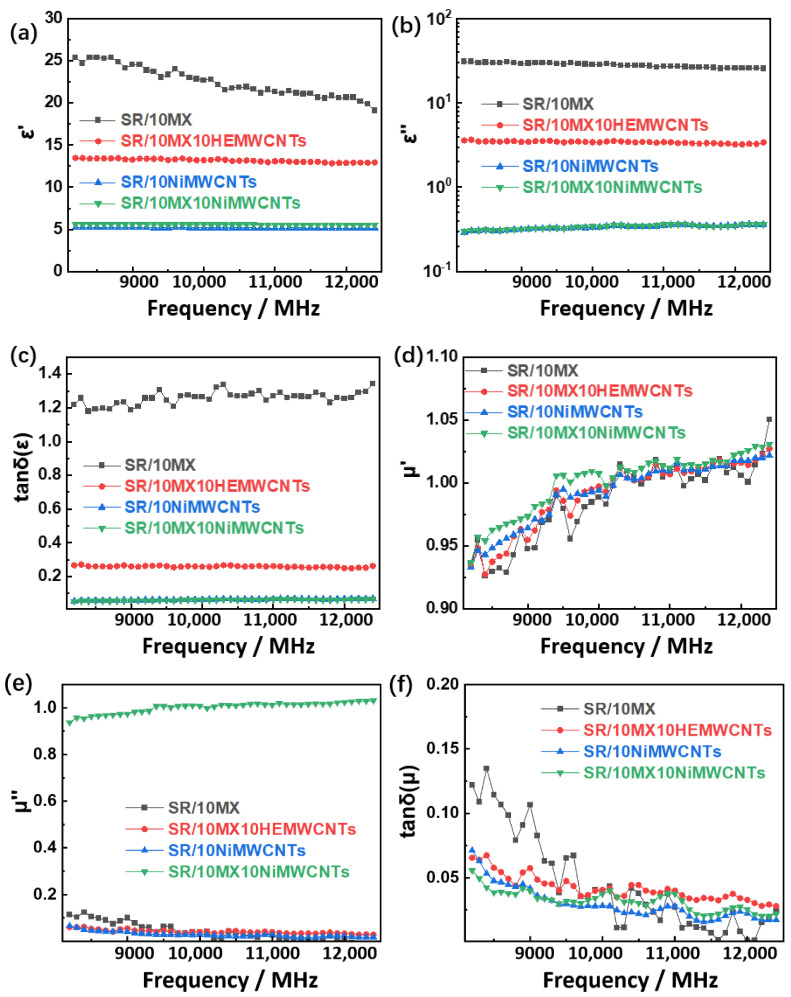
The dielectric and magnetic properties of the silicone rubber/multi-walled carbon nanotubes/MXenes composites: (**a**) dielectric constant (ɛ′-real part); (**b**) dielectric constant (ɛ″-imaginary part); (**c**) dielectric loss factor; (**d**) magnetic permeability (μ′—real part); (**e**) magnetic permeability (μ″—imaginary part); and (**f**) magnetic loss factor.

As shown in Figure 5a, the dielectric constant (ɛ′-real part) of the sample SR/10MX shows a downward trend while the others are stable, and no obvious change of the ɛ″-imagery part was observed for any of them with the increase of the frequency from 8200 MHz to 12,400 MHz in Figure 5b. While the chemical energy kept up with the change of the electric field, the dipole orientation polarization response was not timely to a lower dielectric constant in high frequency for SR/10MX. However, the dielectric constants (ɛ′-real part) of the other three samples were basically stable, and the ɛ′-real part of the latter two with the nickel-added multi-walled carbon nanotubes was around five, and the dielectric constant (ɛ′-real part) of the SR/10MX10HEMWCNTs was fourteen. Both the real part and the imaginary part of the dielectric constant of the SR/10MX were significantly reduced by the highly electric conductive multi-walled carbon nanotubes.

The dielectric loss factors of the three samples SR/10MX10HEMWCNTs, SR/10NiMWCNTs, and SR/10MX10NiMWCNTs were consistent with the change law of the dielectric to be constant, stable, and low, mainly because the dielectric loss of the samples predominantly came from the contribution of atomic polarization and electronic polarization. The dielectric loss factor of the sample SR/10MX also tended to be stable at 1.2, but it was higher than others, as shown in Figure 5c. The SR/10MX composites consumed more energy per unit time under the electric field.

As shown in Figure 5d,e, the magnetic permeability of the four samples exhibited a similar responsiveness in that the magnetic permeability increases as the frequency increases with small vibration. During the low frequency range, the magnetic permeability was less than 1, indicating diamagnetism, but it also showed para-magnetism when the magnetic permeability was higher than 1 after 10,000 MHz.

### 3.6. Electrical Conductivity

**Figure 6:**
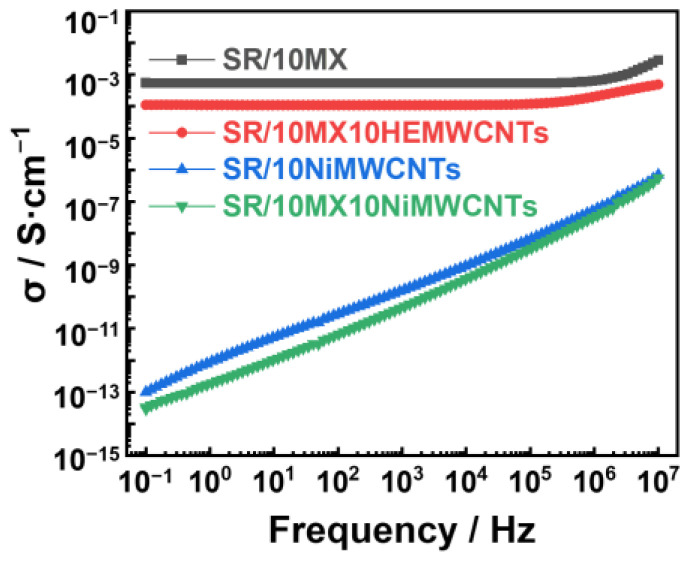
The electric conductivity of the silicone rubber/multi-walled carbon nanotube/MXene composites.

As shown in Figure 6, silicone rubber/highly electric conductive multi-walled carbon nanotube/MXene composites have a good conductivity of more than 10^−4^ S/cm, compared to the composites with nickel-added multi-walled carbon nanotubes. Moreover, the conductivity of the SR/10MX composites was close to 10^−3^ S/cm, indicating that the Ti_3_C_2_MXenes are excellent conductive fillers.

### 3.7. EMI Shielding Effectiveness

**Figure 7:**
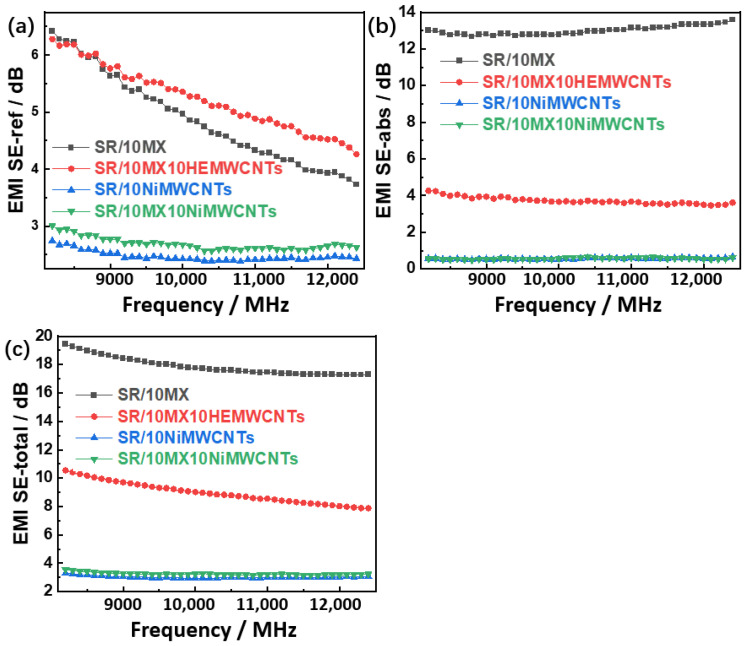
The shielding effectiveness of the silicone rubber/MWCNT/Mxene composites: (**a**) reflection shielding effectiveness; (**b**) absorption shielding effectiveness; and (**c**) total shielding effectiveness.

In the range from 8200 MHz to 12,400 MHz, all the samples with nickel-added multi-walled carbon nanotubes showed a low electromagnetic shielding reflection, absorption, and total effectiveness as shown in Figure 7a–c, respectively. The reflection effectiveness of the SR/10MX10HEMWCNTs markedly decreased with the frequency, but the absorption effectiveness also decreased slightly, while the total effectiveness decreased slightly from 11 dB to 8 dB with the change in frequency. For the SR/10MX composites, the reflection effectiveness clearly decreased with the increase in frequency, but the frequency response behavior of absorption efficiency was low, and the total effectiveness decreased slightly from 20 dB to 17 dB to perform a good electromagnetic shielding performance.

Compared with the SR/10MX sample, the reflection effectiveness of the SR10MX10HEMWCNTs sample was similar, but slightly higher.

### 3.8. Electromagnetic Wave Absorbing Properties

**Figure 8:**
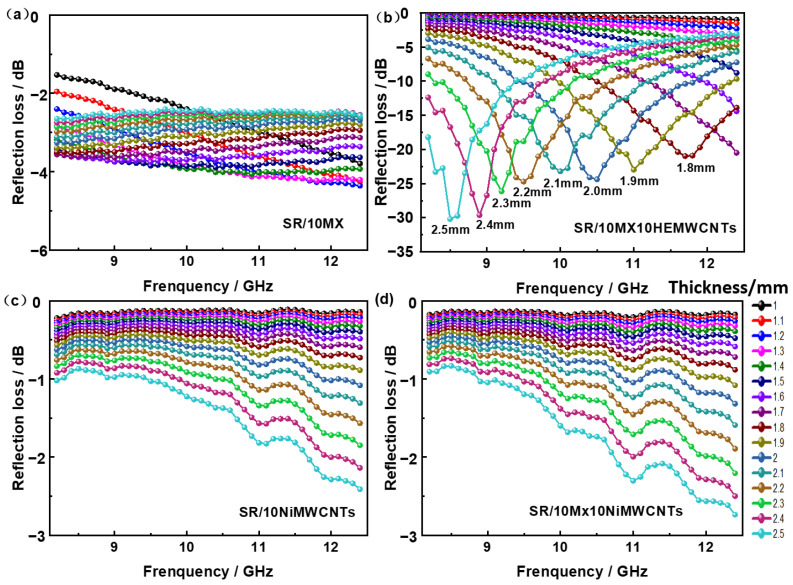
Reflection loss in relation to the thickness of the absorbing materials and the frequency of the incident EM waves of the silicone rubber/multi-walled carbon nanotube/MXene composites: (**a**) SR/10MX; (**b**) SR/10MX10HEMWCNTs; (**c**) SR/10NiMWCNTs; (**d**) SR/10MX10NiMWCNTs.

From Figure 8a,c,d, it can be observed that the sample SR/10NiMWCNTs has a minimum reflection loss of −2.5 dB, while the SR/10MX10NiMWCNTs had a minimum reflection loss of −2.8 dB, and SR/10MX showed a minimum reflection loss of −4.1 dB, respectively. These three composites were far from satisfying the application requirement to be excellent microwave absorbing materials.

As shown in Figure 8b, the minimum reflection loss of the sample SR/10MX10 HEMWCNTs attained −30.19 dB, corresponding to the sample thickness of 2.5 mm, while the minimum reflective loss of the SR/10MX10HEMWCNTs were −21.01 dB, −23.11 dB, −24.44 dB, −23.34 dB, −24.73 dB, −26.41 dB, and −29.63 dB, corresponding to the thicknesses from 1.8 mm to 2.4 mm at the interval gradient of 0.1 mm, respectively.

**Figure 9:**
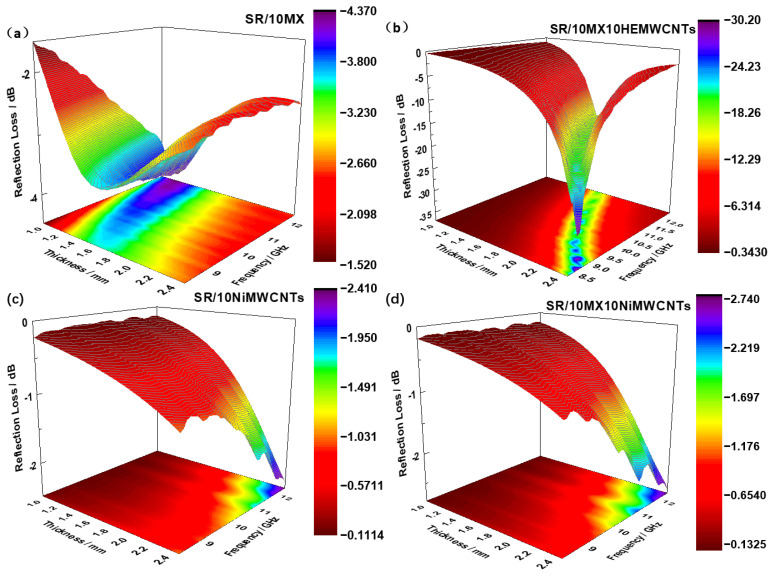
Three-dimensional presentations of reflection loss in relation to the thickness and incident frequency of the electromagnetic wave of the silicone rubber/multi-walled carbon nanotubes/MXene composites: (**a**) SR/10MX; (**b**) SR/10MX10HEMWCNTs; (**c**) SR/10NiMWCNTs; (**d**) SR/10MX10NiMWCNTs.

Figure 9 shows the reflection loss of the silicone rubber/multi-walled carbon nanotube/MXene composites as a function of the thickness and the incident frequency of the electromagnetic wave. In the thickness range from 1.6 mm to 1.8 mm, the SR/10MX sample exhibited the minimum reflection loss band, but the reflection loss was poor, as shown in Figure 9a. The reflection loss of the sample SR/10MX10HEMWCNTs, as shown in Figure 9b, presented a series of sharp peaks with the thickness and the incident frequency of electromagnetic wave. The highly electric conductive multi-walled carbon nanotubes and the accordion-like two-dimensional material Ti_3_C_2_Mxenes constructed a network to a three-dimensional structure combining one-dimensional materials, two-dimensional materials, and the spatial lamination of them, resulting in more wave energy loss and less for reflecting and passing.

The reflection loss of the samples SR/10NiMWCNTs and SR/10MX10NiMWCNTs was not sufficient to be used as absorbing materials, as shown in Figure 9c,d, mainly due to the high metal content in the materials, and that the minimum reflection loss might be shown in a higher frequency.

### 3.9. Dispersion Structure Schematic

**Figure 10:**
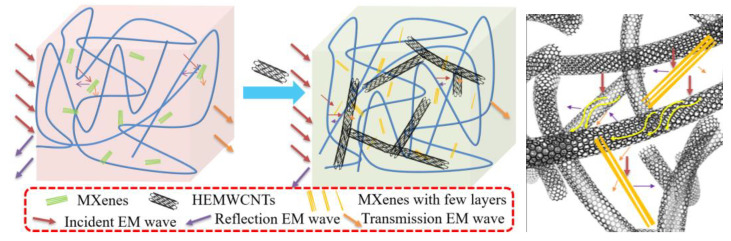
Transformation mechanism illustration from electromagnetic reflection to the absorbing performance of the silicone rubber and accordion-shaped Ti_3_C_2_MXene composites by the highly electric conductive multi-walled carbon nanotubes.

To evaluate the transformation by the HEMWCNTs, a schematic structure for absorbing performance was drawn based on the SEM images and electromagnetic properties, as shown in Figure 10.

Highly electric conductive multi-walled carbon nanotubes benefited from the dispersion of the accordion-like two-dimensional material Ti_3_C_2_MXenes, with few layers to construct a network with a three-dimensional structure combining one-dimensional materials, two-dimensional materials, and the spatial lamination of them.

Without the HEMWCNTs present in the matrix, the electromagnetic wave was reflected and transmitted through an internal reflection absorption by the MXenes with multi-layers in the composites. When the electromagnetic wave has to pass through the silicone rubber composites with the MXenes and HEMWCNTs, the internal reflection becomes more complicated, and more wave energy of electromagnetic is consumed, resulting in less for reflecting and passing.

## 4. Conclusions

The mixed silicone rubber/Ti_3_SiC_2_MXene composites and silicone rubber/HEMWCNT/Ti_3_C_2_MXene composites have been successfully prepared. The silicone rubber composites with Ti_3_SiC_2_MXenes perform a good electromagnetic shielding effectiveness of 20 dB in the X-band for good conductivity of more than 10^−3^ S/cm, display good dielectric properties, and demonstrate a low magnetic permeability with more dielectric loss. With the combination of HEMWCNTs, the composites achieved the transformation from the electromagnetic reflection performance to an excellent absorbing performance. The samples demonstrated good conductivity above 10^−4^ S/cm, a higher dielectric constant, and loss in both the dielectric and magnetic properties to reach a minimum reflection loss of −30.19 dB, thereby showing great potential in the field of EM interference. To solve the electromagnetic pollution, a layer-by-layer constructure is deemed a good option to show excellent electromagnetic interference and wave absorbing performance in that one layer is made of the silicone rubber/Ti_3_SiC_2_MXene composites while another layer is composed of the silicone rubber/HEMWCNTs/Ti_3_SiC_2_MXene composites through a multi-layered alternation.

## Figures and Tables

**Table 1:** Vulcanization parameters of silicone rubber/multi-walled carbon nanotube/MXenes composites.

Items	MH dNm	ML dNm	MH-ML dNm	t_10_ min	t_90_ min
SR/10MX	19.53	10.41	9.12	0.65	4.02
SR/10MX10HEMWCNTs	13.31	6.92	6.39	0.36	2.48
SR/10NiMWCNTs	25.60	3.63	21.97	0.36	2.21
SR/10MX10NiMWCNTs	19.08	4.11	14.98	0.31	1.58

Note: ML-ML—the lowest torque of curing curve; MH—the highest torque of curing curve; MH-ML—the difference between the highest torque and the lowest torque; t_10_—the time corresponding to a 10% increase in torque; and t_90_—the time corresponding to a 90% increase in torque.

## Data Availability

Not applicable.
